# Assessing the effects of mosquito nets on malaria mortality using a space time model: a case study of Rufiji and Ifakara Health and Demographic Surveillance System sites in rural Tanzania

**DOI:** 10.1186/s12936-016-1311-9

**Published:** 2016-05-04

**Authors:** Majige Selemani, Amina S. Msengwa, Sigilbert Mrema, Amri Shamte, Michael J. Mahande, Karen Yeates, Maurice C. Y. Mbago, Angelina M. Lutambi

**Affiliations:** Department of Statistics, University of Dar es Salaam, P. O. Box 35047, Dar es Salaam, Tanzania; Ifakara Health Institute, (IHI), Plot 463, Kiko Avenue, off Old Bagamoyo Road, Mikocheni, P. O Box 78373, Dar es Salaam, Tanzania; Department of Epidemiology & Applied Biostatistics, Kilimanjaro Christian Medical University College, Moshi, Tanzania; Department of Medicine, Queen’s University, 94 Stuart Street, Kingston, Canada

**Keywords:** Space time model, Malaria mortality

## Abstract

**Background:**

Although malaria decline has been observed in most sub-Saharan African countries, the disease still represents a significant public health burden in Tanzania. There are contradictions on the effect of ownership of at least one mosquito net at household on malaria mortality. This study presents a Bayesian modelling framework for the analysis of the effect of ownership of at least one mosquito net at household on malaria mortality with environmental factors as confounder variables.

**Methods:**

The analysis used longitudinal data collected in Rufiji and Ifakara Health Demographic Surveillance System (HDSS) sites for the period of 1999–2011 and 2002–2012, respectively. Bayesian framework modelling approach using integrated nested laplace approximation (INLA) package in R software was used. The space time models were established to assess the effect of ownership of mosquito net on malaria mortality in 58 villages in the study area.

**Results:**

The results show that an increase of 10 % in ownership of mosquito nets at village level had an average of 5.2 % decrease inall age malaria deaths (IRR = 0.948, 95 % CI = 0.917, 0.977) in Rufiji HDSS and 12.1 % decrease in all age malaria deaths (IRR = 0.879, 95 % CI = 0.806, 0.959) in Ifakara HDSS. In children under 5 years, results show an average of 5.4 % decrease of malaria deaths (IRR = 0.946, 95 % CI = 0.909, 0.982) in Rufiji HDSS and 10 % decrease of malaria deaths (IRR = 0.899, 95 % CI = 0.816, 0.995) in Ifakara HDSS. Model comparison show that model with spatial and temporal random effects was the best fitting model compared to other models without spatial and temporal, and with spatial–temporal interaction effects.

**Conclusion:**

This modelling framework is appropriate and provides useful approaches to understanding the effect of mosquito nets for targeting malaria control intervention. Furthermore, ownership of mosquito nets at household showed a significant impact on malaria mortality.

**Electronic supplementary material:**

The online version of this article (doi:10.1186/s12936-016-1311-9) contains supplementary material, which is available to authorized users.

## Background

Evidence about the impact of mosquito net ownership on malaria mortality under routine conditions is limited and contradicting. Several studies have been conducted on the effect of this malaria control intervention [1–5]. Some studies used all cause child mortality as an endpoint [1, 2] while others have focused on indicators of malaria transmission [3, 6] and on changes in treatment outcomes [1, 3]. Other studies have shown a decrease in all-cause mortality associated with insecticide-treated nets (ITN)ownership [1, 2]. Another study found no difference in mortality rates between villages with and without ITNs intervention [5]. However, most of these studies have used a temporal model and only one study identified, on malaria incidence, that considered the spatial–temporal dependency [6]. Therefore, there is a need to assess the effect of mosquito net ownership on malaria mortality in Tanzania.

Insecticide-treated nets (ITNs) have been an effective intervention for preventing malaria transmission by reducing the chances that an individual would be bitten by an infective Anopheles mosquito [7, 8]. Several studies on ITN efficacy or effectiveness conducted have shown continued ability of ITNs protection against malaria transmission [9–11]. Moreover, studies on pregnancy outcome showed that use of bed nets could reduce miscarriages and stillbirths by 33 % and by 23 % in placental parasitaemia [12]. Overall, the use of ITNs have been effective [1, 7, 13]. Following results from studies on efficacy and effectiveness of ITN use in Tanzania, a market-based national ITN voucher programme to scale up the coverage of bed nets was adopted in 2004 [14]. This programme increased household ownership of at least one ITN from 23 to 92 % between 2004/2005 and 2012 in Tanzania mainland [15].

Long-term evaluation of the impact of malaria prevention and control on malaria mortality is lacking, posing a challenge to malaria endemic countries like Tanzania, where many deaths occur at home [16] where no records are available [17]. Demographic and Health Surveys (DHS) have been used as sources of mortality data but lack the ability to provide causes of deaths. Assumptions about the proportion of malaria deaths to all-cause mortality have been used [4, 18, 19].

Verbal autopsy (VA) is currently an alternative approach that has been used to determine malaria causes of deaths in community-based studies in many of the endemic countries where vital registration data are incomplete or do not exist [20, 21]. VA is an indirect method used in determining the causes of death based on an interview with next of kin or other caregivers. This is done using a standardized questionnaire that obtains information on signs, symptoms, medical history and circumstances preceding death [20]. Studies have evaluated the validity of VA for determining malaria-specific death [22, 23] in HDSS sites. The results concluded that VA methods have an acceptable level of diagnostic accuracy for determining malaria deaths at the community level.

In sub-Saharan African (SSA), VA has been routinely used as a tool to provide information on the burden of the disease using HDSS sites [24, 25]. HDSS has sites established within the INDEPTH network in many SSA countries which continuously collect large amounts of data. The data are collected on spatial and temporal scales and include cause specific deaths using VA, in and out migration, and other demographic information [26]. This study assesses the effect of ownership of mosquito nets on malaria mortality using a Bayesian space time model. Also, the study assessed how environmental factors such as rainfall, temperature and vegetation index affect the impact of mosquito net ownership.

## Methods

### Study area

The study was carried out using data from Rufiji and Ifakara Rural Health and Demographic Surveillance System (HDSS) sites [27, 28]. The sites are primarily rural with majority of the population relying on subsistence farming or fishing. The Rufiji HDSS covers 1813 km^2^ that comprises 33 villages of Rufiji District, Coast region. Ifakara HDSS covers an area of 2400 km^2^ across which covers 25 villages (13 in Kilombero and 12 in Ulanga districts) in Morogoro region south-eastern part of the country. These two HDSS sites were selected because they are among the HDSS sites, which continuously collect large amounts of data longitudinally on defined geographical areas for malaria specific cause of death in Tanzania. Furthermore, these HDSS sites continue to have high malaria prevalence. For example, microscopy estimates for health facilities in 2012 in Rufiji were 19.2 % in high season and 7.2 % in low season, and, in Ifakara they were 9.4 % in high season and 4.2 % in low season 4.2 % [29]. In these two HDSS, malaria is the major contributor among deaths in children below 5 years of age [30, 31].

### Study design and data collection

**Fig. 1:**
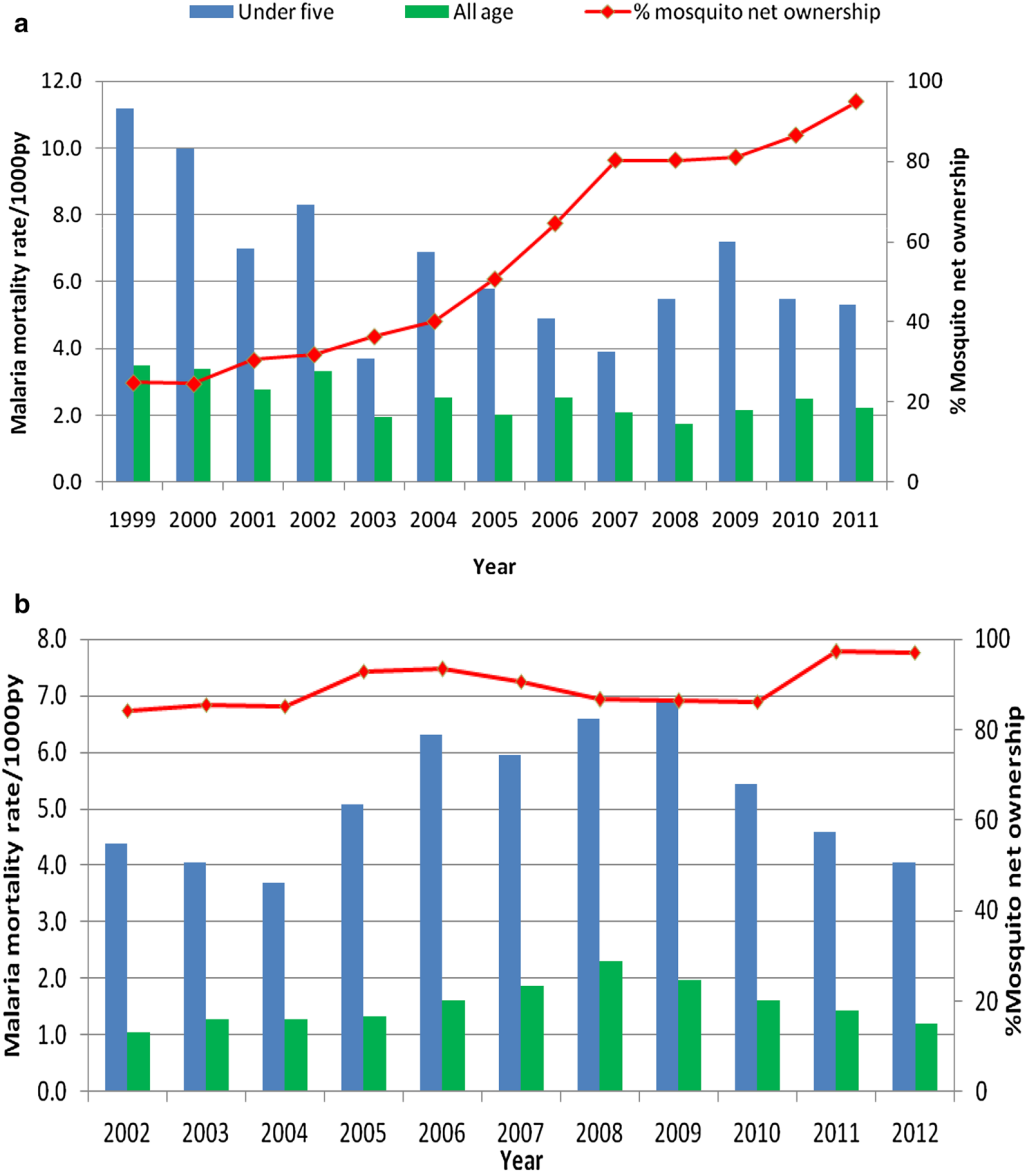
Malaria mortality rates and percentage of households with at least one mosquito nets in Rufiji and Ifakara HDSS. **a** For Rufiji HDSS; **b** for Ifakara HDSS

The analysis used longitudinal data collected from Rufiji and Ifakara HDSS sites. Yearly malaria deaths were extracted from the Rufiji and Ifakara HDSS database covering a period of 1999–2011 and 2002–2012 respectively. The two HDSS sites have consistently been recording pregnancies, pregnancy outcomes, deaths and migrations by visiting households once every 4 months since 1997 in Ifakara HDSS and 1998 in Rufiji HDSS. Also, the HDSS sites collect information on socioeconomic status including household ownership of mosquito nets once per year during visit round of update. The mosquito nets in this study include untreated and treated nets (ITNs). The study period for this particular study covers the social marketing and national scale up of ITNs in the country [32]. The mosquito net ownership in HDSS sites has been increased over the years. In Rufiji HDSS the ownership of at least one mosquito net increased from 25 % of households in 1999 to 95 % in 2011 while in Ifakara HDSS the ownership of at least one mosquito net has been high coverage since 2001 (Fig. 1). The details on how data collection, management and credibility in HDSS are discussed in detail elsewhere [27, 28].

### Verbal autopsy procedure

The WHO and INDEPTH network [33] standardized VA questionnaire was adapted and used for data collection on causes of death. In the HDSS, deaths were captured during sequential rounds of data collection updates. Then the trained HDSS field interviewers visited the deceased’s home after a grieving period to administer a verbal autopsy questionnaire. An interview was administered to relatives or caregivers who were closely associated with the deceased during the period leading to his or her death. The questionnaire assessed the identity of the deceased and established the sequence of events leading to death, including symptoms and signs of the illness before death. Verbal autopsy was carried out since 1998 in Rufiji HDSS and 2002 in Ifakara HDSS. The verbal autopsy forms are independently reviewed by two physicians according to a list of causes of death based on the 10th revision of the international classification of diseases (ICD-10). A third physician is asked to code the cause of death in the case of discordant results. If there is disagreement among the three physicians, the death is coded as “undetermined” cause [34]. Causes of death (main, immediate, and/or contributing) are coded to be consistent with the ICD-10 [35]. Malaria deaths are coded as direct where malaria is the underlying cause of death or indirect where malaria is one of several diseases leading to death but where the death is attributed by a different cause [13]. In this analysis direct and indirect malaria deaths were included.

### Confounding variables

The analysis used mean rainfall, mean temperature, mean normalized difference vegetation index (NDVI) and age as confounding variables in assessing the effect of mosquito net ownership on malaria mortality.

Climate data includes monthly mean rainfall and temperature (maximum and minimum) with these measures obtained from Tanzania Meteorological Authority (TMA) head office in Dar es Salaam. TMA provides meteorological services, weather forecasts, climate services, and warnings including daily forecast information for each region in Tanzania [36]. The climate data are collected through the different gauges located in different stations in each district. In Rufiji District, Utete, Ikwiriri and Kibiti weather station records climate data. Kibiti weather station data were used for Rufiji HDSS site and the Kilombero Agricultural Training and Research Institute (KATRIN) weather station data were used for villages in Ifakara HDSS for Kilombero district. Other villages used Mahenge weather station data for villages located in Ulanga district.

Remote sensing data,which included normalized difference vegetation index (NDVI)was extracted from moderate resolution imaging spectroradiometer (MODIS) on board NASA’s terra satellite. The vegetation index was processed from MODIS (MOD13A3) using monthly composite images at a 1 × 1 km resolution. Vegetation indices are used for global monitoring of vegetation conditions and are used in products displaying land cover and land cover changes. ERDAS Imagine software version 10.1 was used for processing the satellite images, and ArcGIS version 10 was used for spatial analyses. For each village within the study area, mean NDVI were calculated each year to link with malaria mortality for each village. The village boundaries were overlaid with the raster environmental data (i.e., MODIS) and calculated the weighted mean value for each village based on the proportion of the village area.

### Data processing and analysis

**Fig. 2:**
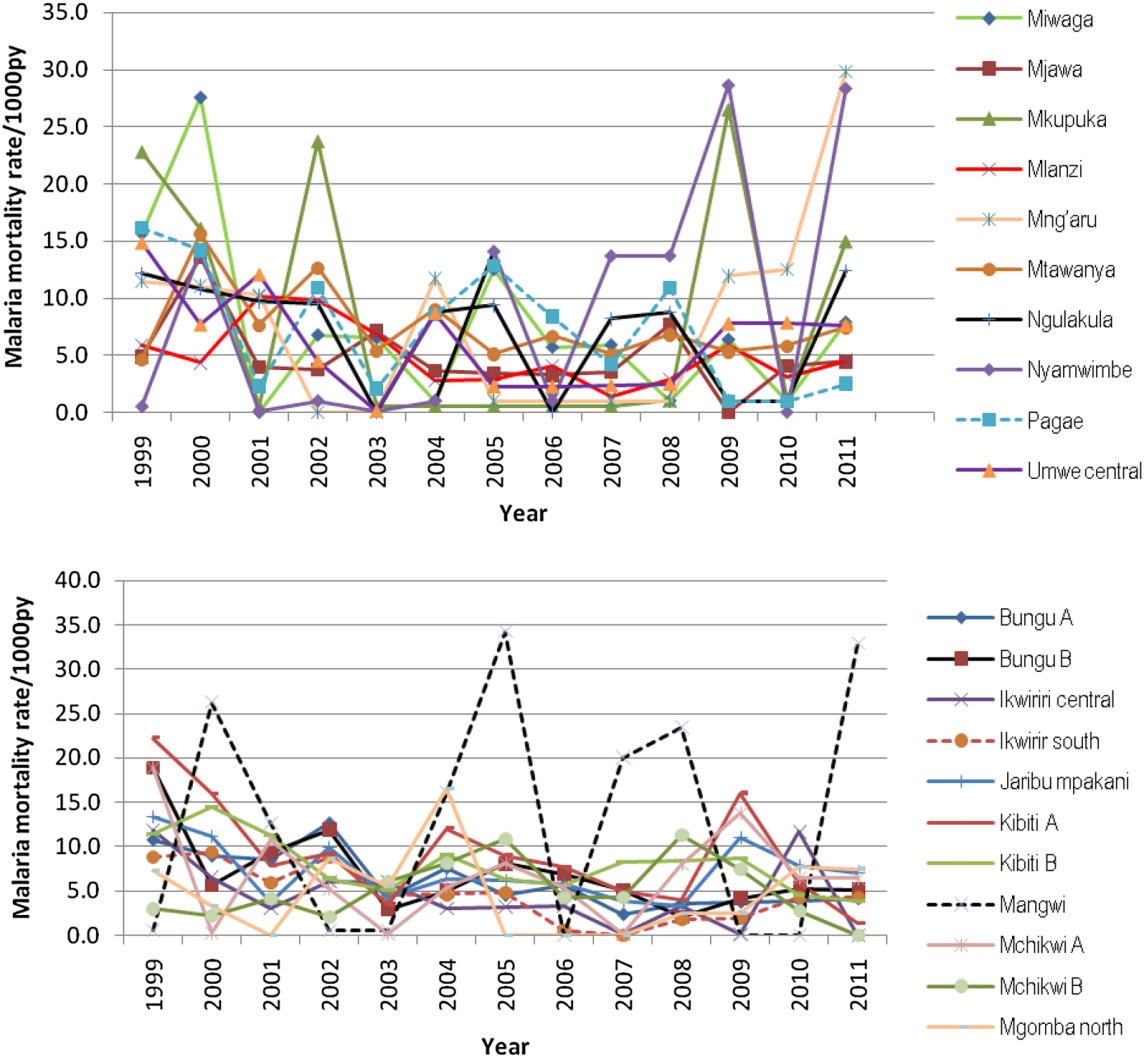
Annual malaria mortality rate trends for under-five by village in Rufiji HDSS

**Fig. 3:**
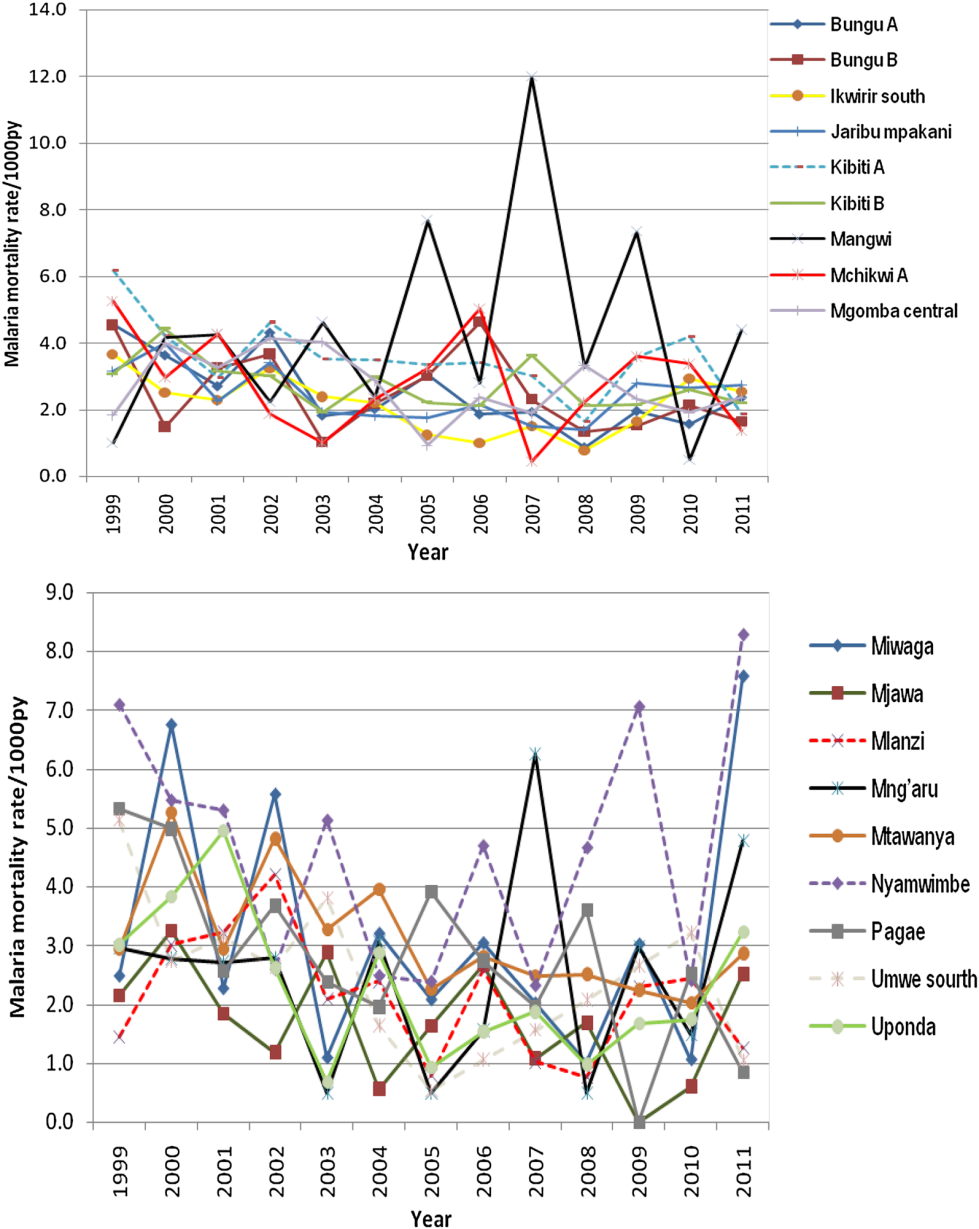
Annual malaria mortality rate trends for all age by village in Rufiji HDSS

**Fig. 4:**
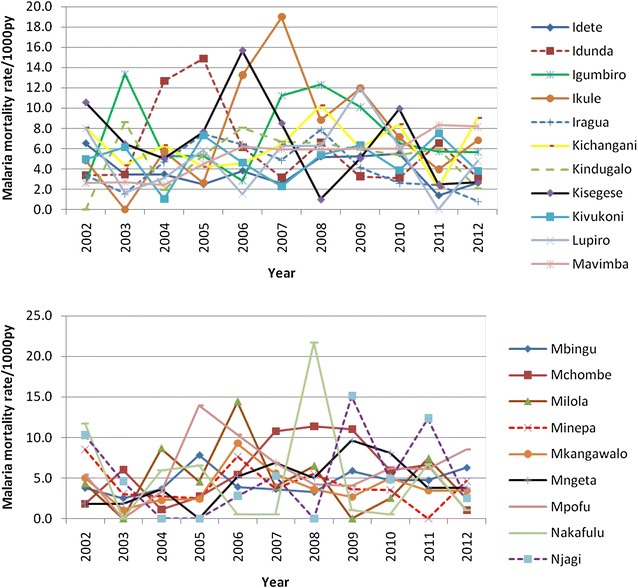
Annual malaria mortality rate trends for under-five by village in Ifakara HDSS

**Fig. 5:**
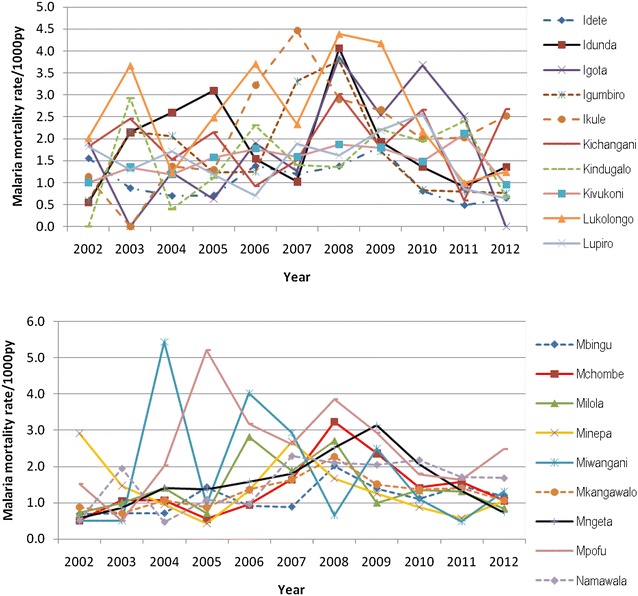
Annual malaria mortality rate trends for all age by village in Ifakara HDSS

The number of malaria deaths in each village were extracted from the HDSS database and aggregated yearly in order to link with mosquito net ownership which is also surveyed yearly in the HDSS sites. The percentages of household’s mosquito net ownership at village level were calculated each year. Time at risk (person-years) contributed by each person was calculated from 1st January for each year until exit. Exit from the study was due to migration (outside the HDSS area), death or end of the study. In a case where a person migrated to a different household location within the study area, time at risk was computed separately for new location and added to the total time at risk. The outcome of interest is total yearly death due to malaria at village level for specific age groups. The age was categorized into under five and five and above. The outcome variable was aggregated at village level to capture heterogeneity for mortality in the study area [37, 38]. The malaria mortality rates were calculated by dividing the number of deaths by the person-years of observation and were expressed per 1000 person-years (py). Figures 2, 3, 4, and 5 display malaria mortality by village in each HDSS site.

Monthly environmental and climate data were aggregated and averaged to obtain mean values to link with the mosquito net ownership and malaria death data. Mean annual rainfall (millimetre), mean annual temperature (^°^C), and mean annual NDVI were used as confounder variables because they are associated with malaria transmission [39, 40]. The percentage of households ownership at least one mosquito netat household was used also as main explanatory variable.

In order to highlight spatio-temporal trends of malaria mortality in the study areas, direct estimates of malaria mortality rates were calculated. The malaria mortality rates were calculated by dividing the number of deaths by the person-years of observation and were expressed per 1000 person-years (py).

### Space time hierarchical model

This study propose a spatial and temporal hierarchical model that considers the effects of predictors variables as described in detail [41]. These type of models usually have to model data, processes, and parameters [42]. The study used space time models that includes spatial and time random effects as described [43]. Covariate informations considered in this study for village *i* at year *j* were mosquito net ownership as exposure variable and confounder variables (rainfall, temperature and NDVI). For this study *i* the number of village in the study, *I* = 33 for Rufiji and 25 for Ifakara HDSS and *j* for years, *J* = 13 for Rufiji HDSS and *J* = 11 for Ifakara HDSS.

The processes are modelled by common specifications where both spatial component and temporal random time effects as first autoregression AR (1) are considered (see Additional file 1). In a broad view, random effects specifications for the spatial random terms include the unstructured noise term that follows a normal distribution and structure term which modelled with the set of villages that adjacent to other villages by weight of neighboring village. Weight is considered to be 1 when villages *i* and *j* share the same boundary and 0 otherwise. The prior distribution of the random effect term is a conditional autoregressive prior used to model the spatial dependence. The temporal random effect determines the prior distribution consider a first order auto-regression AR (1) given by the partial autocorrelation function. The distribution of the interaction term is characterized by a precision matrix obtained as the Kronecker product of the precisions of unstructured and structured noise terms [44].

Ownership of at least one mosquito net was considered as the major malaria control strategy in this study. Additional confounding variables incorporated in the space time model included annual mean rainfall, and average annual temperature, and mean annual NDVI. Models considered included a non-spatial and temporal effect model (*M*_*1*_), a model with spatial and temporal random terms (*M*_*2*_) and a model with spatial and temporal random terms, and interaction (*M*_*3*_) (see Additional file 1). Model (*M*_*2*_) and (*M*_*3*_) included spatial random effects which arising from a Gaussian stationary process with covariance matrix capturing correlation between any pair of villages as a function of their distance. Additional models were considered to check for multi-colinearity using variance inflation factors for environmental variables. The model was checked for statistical interactions between mosquito net ownership and confounder variables.

In this analysis, R programming language (R Development Core Team, 2009) using the INLA package was used to fit three models defined *M*_1_ to *M*_3_. Latent models available in INLA package such as Besag model [45], an independent random noise (iid) model, and first order auto-regression AR (1) [46] were used to fit models to data. Besag model is frequently used approach to spatial disease modelling in small areas [45]. INLA package is a computational approach used to approximate Bayesian inference based on an efficient combination of Laplace approximations and numerical integration. Unlike Markov Chain Monte Carlo (MCMC), the INLA method does not sample from the posterior distribution [47]. Posterior distributions are approximated with a closed form expressions, reducing convergence and mixing problems. INLA is suitable for Bayesian hierarchical models for large number of unknown parameters that follow a Gaussian Markov random field and a small number of hyper-parameters.

### Model selection

Considering the dynamic nature of malaria, an important issue was whether ownership of mosquito nets has an impact on malaria death. Similar to Arab et al. [30], deviance information criterion (DIC) was used to assess model goodness of fit and selection for best model that produced lower values of the DIC. DIC is a generalization of the Akaike and Bayesian information criterion [48]. Model estimates were then exponentiated to obtain the incidence rate ratios (IRR) for each predictor.

### Ethical approval

The Ethical clearance was granted by the Ifakara Health Institute (IHI)’s Institutional Review Board (IRB), Tanzania and Medical Research Coordinating Committee (MRCC) of the National Institute for Medical Research (NIMR) for the establishment of Rufiji and Ifakara HDSS. For each household visit, verbal consent was sought from the respondent.

## Results

Table 1 shows the annual malaria mortality rate in Rufiji and Ifakara HDSS. In Rufiji HDSS, malaria mortality rate decreased from 11.2 per 1000 person-years in 1999 to 5.3 per 1000 person-years in 2011 for under five children age, and from 3.5 per person-years in 1999 to 2.2 per 1000 person years in 2011 for all ages. In Ifakara HDSS, results show the decrease in malaria mortality rate from 6.9 per 1000 person-years in 2009 to 4.0 per 1000 person-years in 2012 for under five children age. Furthermore, the malaria mortality rate for all age decreased from 2.3 per person-years in 2008 to 1.2 per 1000 person years in 2012. However, there was a delay in the decrease in mortality due to malaria rate for both under five children and all age in Ifakara HDSS as shown in Table 1.

**Table 1:** Malaria mortality trend and environmental factors in Rufiji and Ifakara HDSS

Year	Rufiji HDSS								
	Under-five			All age			Environmental factors		
	Person-years (py)	Malaria deaths	U5MMR/1000	Person-years (py)	Malaria deaths	AMMR/1000	Annual mean rainfall	Annual mean temperature	Annual mean NDVI
1999	10,637.4	119	11. 2	65,171.04	229	3.5	1032.2	312.1	0.58
2000	13,211.3	132	10	79,039.43	268	3.4	1020.8	314.3	0.55
2001	13,846.9	97	7	82,200.12	229	2.8	806.5	316.9	0.55
2002	14,472.9	120	8. 3	84,396.51	282	3.3	1210.0	319. 0	0.54
2003	14,997. 9	55	3. 7	85,425.96	167	2	664. 8	328. 4	0.46
2004	14,812.8	102	6.9	85,600.83	218	2.5	1171.1	321.1	0.43
2005	14,603.8	85	5.8	86,047.84	173	2	547.6	318.7	0.60
2006	14,950.7	74	4.9	86,943.32	220	2.5	935.3	315.4	0.61
2007	14,839.9	58	3.9	87,561.88	183	2.1	732.5	319.4	0.54
2008	14,264.6	78	5.5	86,687.00	151	1.7	898.5	315.4	0.51
2009	13,631.7	98	7.2	85,229.04	185	2.2	730.5	321.9	0.47
2010	13,065.5	72	5.5	81,454.82	205	2.5	601.7	323.1	0.54
2011	12,947.8	69	5.3	85,374.06	189	2.2	652.6	324.8	0.54
*Ifakara HDSS*									
2002	12,095.8	53	4.4	71,744.7	74	1.0	2033.7	244.9	0.49
2003	12,309.4	50	4.1	72,269.4	92	1.3	1194.4	256.0	0.47
2004	12,773.2	47	3.7	765,422.0	97	1.3	1826.9	253.3	0.47
2005	13,027.4	66	5.1	78,772.8	104	1.3	1378.1	258.1	0.58
2006	13,953.0	88	6.3	84,497.7	136	1.6	1862.7	253.9	0.49
2007	14,938.3	89	6.0	88,827.5	166	1.9	1580.1	256.2	0.56
2008	16,219.5	107	6.6	95,463.7	221	2.3	1721.5	251.7	0.55
2009	16,912.0	117	6.9	101,121.7	200	2.0	1519.5	258.7	0.52
2010	19,119.8	104	5.4	114,664.3	184	1.6	1565.7	245.4	0.59
2011	19,777.0	91	4.6	120,382.7	173	1.4	1842.8	256.6	0.58
2012	19,814.1	80	4.0	123,806.0	149	1.2	1217.0	253.6	0.50

*U5MMR* malaria mortality rate for under-five; *AMMR* malaria mortality rate for all age

Figure 1 indicates the percentages of households that owned at least one mosquito net and malaria mortality rate in the two HDSS sites. The percentages have been increased while the malaria mortality rate decreased in Rufiji HDSS. Ifakara HDSS has high percentage of households with at least one mosquito net ownerships since 2002.

Malaria mortality rate by village in the selected villages in HDSS sites are shown in Figs. 2, 3, 4 and 5. The results show that in most of the villages, malaria mortality rates have been decreasing and few villages show high malaria mortality for exception years. Malaria mortality rate for under-five children were high in Mangwi village in 2005 and 2011, Nyamwimbe in 2007 and 2011 compared to other years and villages (Fig. 2). In 2005 and 2007 there was high malaria mortality in Mangwi and Nyamwimbe in 2011 for all age (Fig. 3). In Ifakara, HDSS, malaria mortality declined from 2008 to 2012 for both all age and under-five children. High malaria mortality for under five was observed in Nakafuru in 2008 and Igota village in 2011 (Fig. 4). In addition, higher malaria mortality for all age was observed in 2004 in Miwangani village and Mpofu village in 2005 (Fig. 5).

Three space time models were implemented for malaria mortality and ownership of mosquito nets and environmental factors. Table 2 shows the deviance information criterion for fitted three models in Rufiji HDSS. Based on DIC values, the model with spatial and temporal random effect terms performed best among the models considered (DIC = 1880. 076), followed by the model with spatial and temporal interaction with DIC = 1902.217. The model without spatial and temporal effect had the worst performance (DIC = 1933.614). Also these models were fitted to malaria mortality for under five children, model with spatial and temporal random effect performed better compared to other two models (DIC = 1483.748). Based on these results, it is clearly observed that including spatial and temporal random effect terms is necessary.

**Table 2:** Adjusted estimated effect of ownership of mosquito net on malaria mortality in three models for Rufiji HDSS

	M_1_	M_2_	M_3_
	IRR (95 % CI)	IRR (95 % CI)	IRR (95 % CI)
All age			
Intercept	0.004 (0.00, 0.180)	0.0002 (0.0, 0.170)	0.0002 (0.0, 0.175)
Mosquito nets	0.959 (0.944, 0.975)	0.948 (0.917, 0.977)	0.950 (0.919, 0.977)
Annual rainfall	1.047 (1.024, 1.071)	1.066 (1.031, 1.107)	1.066 (1.031, 1.106)
Average temperature	0.998 (0.986, 1.009)	1.007 (0.986, 1.031)	1.006 (0.986, 1.029)
Mean NDVI	1.045 (0.985,1.109)	1.058 (0.974, 1.146)	1.059 (0.976, 1.147)
Spatial random effect		21.89 (6.82, 57.92)	22.16 (6.89, 59.28)
Temporal random effect		80.26 (11.49, 265.0)	120.96 (13.44, 475.23)
Spatial–temporal random effect			24,453.3 (1988.4, 97, 438.8)
DIC	1933.614	1880.076	1902.217
Under five			
Intercept	2.294 (0.01, 72.539)	0.151 (0.0, 18.81)	0.160 (0.0, 14.161)
Mosquito nets	0.951 (0.929, 0.973)	0.946 (0.909, 0.982)	0.946 (0.910, 0.982)
Annual rainfall	1.037 (1.003, 1.072)	1.056 (1.008, 1.110)	1.054 (1.007, 1.107)
Average temperature	0.982 (0.965, 0.998)	0.989 (0.962, 1.100)	0.989 (0.963, 1.018)
Mean NDVI	1.010 (0.926, 1.102)	1.030 (0.934, 1.137)	1.030 (0.934, 1.137)
Spatial random effect		28,708.6 (3481.2, 89,382.7)	28,499.5 (3476.5, 88, 897.6)
Temporal random effect		44.87 (7.71, 139.67)	54.73 (8.59, 180.77)
Spatial–temporal random effect			18,370.02 (18,242.8, 66, 475.6)
DIC	1504.408	1483.748	1508.173

The effect of rain was estimated for every 100-mm increase in mean total annual rainfall, and the effect of mosquito net for every 10 % increase in household ownership. The effect of NDVI was estimated for every 0.1 increase in mean NDVI

*IRR* incidence rate ratio; *M*_*1*_ model without spatial and temporal random terms; *M*_*2*_ model with spatial and temporal random terms; *M*_*3*_ model with spatial and temporal random terms and interaction

Table 2 also shows that mosquito net ownership at household was associated with malaria mortality in Rufiji HDSS. The results in Table 2 show that an increase of 10 % ownership of at least one mosquito net at households in village is associated with (IRR = 0.948, 95 % CI: 0.917–0.977) 5 % decrease of malaria risk for all age per year and (IRR = 0.946, 95 % CI: 0.909–0.982) 5 % for under five children per year. The results show an increase of 100 mm for rainfall corresponds approximately to 6.6 % (IRR = 1.066, 95 % CI: 1.031–1.107) increase of malaria risk for all age per year. For under-five children, increase of 100 mm for rainfall corresponds to increase of (IRR = 1.056, 95 % CI: 1.031–1.107) 5. 6 % in malaria risk for under-five children per year.

Table 3 shows incidence rate ratio for the regression coefficients in three models for Ifakara HDSS with DIC. The model with spatial and temporal random effect terms performed best among the models considered (DIC = 1226.043) and (DIC = 1047.046) for all age and under five rates, respectively. Model comparison showed that model with spatial and temporal random effect had a small DIC value and therefore was the best fitted model.

**Table 3:** Adjusted estimated effect of ownership of mosquito net on malaria mortality in three models for Ifakara HDSS

	M_1_	M_2_	M_3_
	IRR (95 % CI)	IRR (95 % CI)	IRR (95 % CI)
All age			
Intercept	0.0005 (0.00, 0.007)	0.0203 (0.00, 2.376)	0.0215 (0.00, 2.495)
Mosquito nets	0.885 (0.830, 0.879)	0.879 (0.806, 0.959)	0.887 (0.812, 0.970)
Annual rainfall	1.015 (0.999, 1.031)	1.008 (0.986, 1.030)	1.006 (0.983, 1.027)
Average temperature	1.007 (0.996, 1.017)	0.993 (0.975, 1.009)	0.993 (0.974, 1.009)
Mean NDVI	1.066 (1.016, 1.118)	1.031 (0.972, 1.0935)	1.022 (0.961, 1.086)
Spatial random effect		87.4 (11.7, 347.2)	82.9 (10.9325.9)
Temporal random effect		25.7 (5.3, 72.4)	24.7 (5.1, 69.9)
Spatial–temporal random effect			18,607.1 (1277.5, 67, 205.5)
DIC	1305.891	1226.043	1241.542
Under five			
Intercept	0.003 (0.00, 0.055)	0.010 (0.00, 1.208)	0.011 (0.00, 1.428)
Mosquito nets	0.883 (0.812, 0.961)	0.899 (0.816, 0.995)	0.904 (0.818, 1.003)
Annual rainfall	1.016 (0.995, 1.038)	1.013 (0.988, 1.039)	1.012 (0.987, 1.038)
Average temperature	1.007 (0.993, 1.021)	0.999 (0.981, 1.017)	0.999 (0.980, 1.017)
Mean NDVI	1.048 (0.983, 1.117)	1.026 (0.956, 1.099)	1.020 (0.949, 1.096)
Spatial random effect		27,728.7 (3108.7, 87, 940.7)	27,988.6 (3083.7, 87,704.5)
Temporal random effect		54.137 (6.794, 186.754)	47.28 (6.32, 160.28)
Spatial–temporal random effect			18,164.1 (1237.6, 65, 880.6)
DIC	1061.281	1047.046	1062.05

The effect of rainfall was estimated for every 100-mm increase in mean total annual rainfall, and the effect of mosquito net for every 10 % increase in household ownership

The effect of NDVI was estimated for every 0.1 increase in mean NDVI

*IRR* incidence rate ratio; *M*_*1*_ model without spatial and temporal random terms; *M*_*2*_ model with spatial and temporal random terms; *M*_*3*_ model with spatial and temporal random terms, and interaction

The results show that ownership of at least one mosquito net at household was associated with malaria mortality. In Table 3, the results show that an increase of 10 % ownership of at least one mosquito net at household in village is associated with (IRR = 0.879, 95 % CI: 0.806–0.959) 12 % and (IRR = 0.899, 95 % CI: 0.816–0.995) 10 % decrease of malaria risk for all age and under-five children per year respectively. Also for an increase of 100 millimetres for rainfall there is approximately (IRR = 1.008, 95 % CI: 0.986–1.030) 1 % and (IRR = 1.018, 95 % CI: 0.988–1.039) 1.3 % increase of malaria risk for all age and under-five children per year, respectively. All models (Table 3) indicated mean annual temperature, and mean NDVI were positive predictors of increased annual malaria mortality in contrast to ownership of mosquito nets which had a reducing effect. The model without spatial and temporal effect underestimated the malaria mortality risk. The effect of mosquito net ownership was not affected by the effect of environmental factors (rainfall, temperature and mean NDVI). There was no statistical significance for interaction of mosquito net ownership and environmental factors in the study areas.

The posterior mean risks, represent smoothed values of the raw standardized malaria mortality ratios and, therefore, give a smoothed picture of what is going on with the distribution of malaria mortality in the study area. Model two with spatial and temporal random effect used to predict year 2011 for Rufiji HDSS and 2012 for Ifakara HDSS. The observed malaria mortality and predicted malaria mortality for the 2011 in Rufiji HDSS and 2012 in Ifakara HDSS respectively using model with spatial and temporal random terms (see Additional files 2, 3). The results from each of the Additional files is that the villages with high observed malaria mortality tend to have a high predicted malaria mortality risk; this is consistent with the results in Figs. 2, 3, 4, and 5 for malaria mortality rate.

## Discussion

The malaria mortality rate in the study area decreased by 52.7 and 37.1 % for under-five and all age respectively between 1999 and 2011 in Rufiji HDSS. Also, malaria mortality rate decreased from 2009 in Ifakara HDSS. The space time model suggests that mosquito net ownership is a protective risk for malaria mortality in the study area. The findings show that a 10 % increase in the ownership of at least one mosquito net in the study areas was associated with 6 and 10 % reduction of malaria mortality for under-five children and 5 and 12 % for all ages in Rufiji and Ifakara HDSS, respectively. This result is in line with other previous studies [1, 49] in the same areas which investigated the impact of mosquito net ownership to all cause child mortality. This analysis adds to the existing literature by providing evidence of the effect of mosquito net ownership on malaria mortality in small scale geographic areas; previous studies were predominantly in all cause mortality. The effect of mosquito net ownership in this study was higher in Ifakara HDSS (about 12 % decrease) compared to Rufiji HDSS (5 %). The possible explanation for this is that a number of key malaria control interventions have been implemented in Ifakara HDSS to increase ITN coverage. For example, the KINET project was piloted to promote and subsidize ITNs between 1997 and 1999 [50]. This was also followed subsequently by scale-up nationwide within the frame of the Tanzania National Voucher Scheme (TNVS) [51].

The findings from this study support the continued scale-up of mosquito nets to increase coverage within households in Tanzania. This is an important measure to prevent malaria and is one of the core strategies of World Health Organization’s Roll Back Malaria programme [52]. It also emphasizes the importance of ongoing and future efforts to maintain mosquito net coverage to vulnerable groups and the population as a whole. Furthermore, it also suggests that the massive effort to scale up mosquito net coverage over the past decade has paid off with measurable impacts and that it is possible for health systems to increase coverage of interventions and affect health outcomes over a relatively short period of time. Continued coordinated efforts between local and national governments, international organizations, funding agencies, and researchers are needed to make sure that mosquito nets are reaching all populations at risk for malaria. With the relatively large impact of mosquito nets on child mortality, The findings of this study also support the continued emphasis on malaria control, including the push towards malaria elimination, as a way of improving reduction of malaria deaths at the household and country level.

The findings show an association between rainfall and malaria mortality in under-five age and all ages. The findings of this study are similar to previous assessments of weather related mortality [1, 53, 54]. Rainfall is widely considered to be a major driver of inter-annual variability of malaria incidence in Africa. About 90 % of the deaths that occurred in sub-Saharan Africa are believed to be due to malaria [55]. The possible explanation for this is that rainfall provides breeding sites for mosquitoes and increases the humidity which enhances their survival and therefore increases the spread of the disease [56]. In addition, excess rainfall is known to create considerable flooding which limits access to care and increases the risk of other diseases such as diarrhoea, which in turn increases physical vulnerability and risk of dying from malaria infection, especially in pregnant women and in children under five [57].

Findings of this study indicate that models with spatial and temporal random effect terms performed better for goodness of fit and the effect of mosquito net ownership was higher compared to other models. This shows the necessity for inclusion of spatial and temporal terms in the Bayesian model framework; which is supported by other studies [58–60] that also described the potential use of spatial and temporal terms in the model. The possible explanation is because of the complex dependence patterns over space and over time of the occurrence of malaria deaths, and the inherent large stochastic variability due to rare events. Estimating time trends in each area separately is not efficient enough because it will be difficult to establish a baseline pattern separately for each area.

### Strengths and limitations

This study used datasets from Health and Demographic Surveillance System sites which continuously registered vital demographic events in a geographical defined area. The study findings from health and demographic surveillance systems data provides information to policy makers and program managers which can be translated into policy and practice for targeting malaria control interventions. Although, this study used VA data collected in HDSS sites, few studies have used VA data for investigating malaria cause specific mortality [20, 61, 62], verbal autopsy has great potential for countries like Tanzania where more people die outside of health facility care where no records are available. Also, VA has been shown to provide the best results to obtain the specific causes of death in most of SSA [63] and widely used [22].

This study has some limitations that need to be considered in interpreting the findings. First, presence of at least one mosquito net was considered as a proxy for use of mosquito net in the household. Information about exact use of the mosquito net was not collected during the study because significantly more investment is required for capturing this information. Secondly, there is a potential risk of misclassification about the causes of death where the sensitivity and specificity of the VA technique is relatively low for assessing cause of death and this may lead to underestimate or overestimate for malaria death. Thirdly, there are other possible factors associated with malaria deaths that were not used as confounders in this study and were not available in HDSS database, such as the availability of anti-malarial treatment.

## Conclusions

This study used Bayesian framework modelling to assess the effect of mosquito net ownership on malaria mortality in rural Tanzania. The proposed hierarchical model takes into account spatial and temporal dependencies effect and environmental factors as confounders. It provides a useful tool for health environmental investigations and other factors. Results demonstrate that the proposed modelling approach is robust and can be useful in understanding the effect of ownership of mosquito net on the malaria mortality. Additionally, the model can be applied to analyze spread of other infectious diseases and can be extended to account for other factors such socio-economic factors as well as other important factors such as access to health, information on drug availability and drug resistance.

Reduction in mortality due to malaria requires more attention given to climate change such as rainfall and other non- climatic factors that affect malaria deaths such as ownership of mosquito nets. Ownership and use of mosquito nets should be a continuous strategy in the study areas and Tanzania as a whole for better prevention of malaria transmission and death.

## Additional files

10.1186/s12936-016-1311-9 Appendix.
10.1186/s12936-016-1311-9 Observed and predicted malaria mortality rate using model 2 for 2011in Rufiji HDSS. A1 Observed malaria mortality rate for under-five, A2 predicted malaria mortality rate for under-five, B1 Observed malaria mortality rate for all age and B2 Predicted malaria mortality for all age.
10.1186/s12936-016-1311-9 Observed and predicted malaria mortality rate using model 2 for 2012in Ifakara HDSS. A1 Observed malaria mortality rate for under-five, A2 predicted malaria mortality rate for under-five, B1 Observed malaria mortality rate for all age and B2 Predicted malaria mortality for all age.
