# Supplementary material for: Assessing the effects of mosquito nets on malaria mortality using a space time model: a case study of Rufiji and Ifakara Health and Demographic Surveillance System sites in rural Tanzania
Source: Malar J. 2016 May 4;15:257. doi: 10.1186/s12936-016-1311-9 (PMC4857246; doi:10.1186/s12936-016-1311-9)
Supplement: Supplementary file 1 — 10.1186/s12936-016-1311-9 Appendix. [file 12936_2016_1311_MOESM1_ESM.docx]

**Appendix**

Direct estimates of the malaria mortality rates were obtained as:

$$\theta_{ij}=\frac{Y_{ij}}{P_{ij}}$$

Where $\theta_{ij}$ is the malaria mortality rate at time *j* in village *i*, $Y_{ij}$ is the number of malaria deaths at time *j* in village *i*. $P_{ij}$ represents the number of person-years in village *i* at time *j* and represent the exposure to risk. Let $Y_{ij}$ be the yearly number of malaria death in village *i* for the year *j*, *i*=1 . . . *I* and *j*=1. . . *J*, data model with a Poisson distribution given by:

$Y_{ij}\sim Poisson(P_{ij}\theta_{ij}$) =Poisson ($\mu_{ij}$)

The space time model that includes spatial and time effects defined as:

$$\log\left( \theta_{ij} \right)=\alpha_{0}+\beta X_{ij}+\sum_{k=1}^{3} \beta_{k}X_{kij}+S_{i}+T_{j}+{ST}_{ij}$$

Where $\theta_{ij}$denotes the malaria risk for village *i* in time *j*, and $\mu_{ij}$ denotes the ratio of mean number of malaria death and population. *S* = spatial term(s), *T*= temporal terms(s), *ST* = space-time interaction. Covariate information for village *i* at year *j* is denoted by $X_{ij}$, $\beta$ are regression coefficient for mosquito net ownership, with $\beta_{k}$as the corresponding regression coefficients for k confounder variables (rainfall, temperature and NDVI

The following models are considered:

$\log\left( \theta_{ij} \right)=\alpha_{0}+\beta X_{ij}+\sum_{k=1}^{3} \beta_{k}X_{kij}$ (Non spatial or temporal) *M_1_*

$\log\left( \theta_{\mathrm{ij}} \right)=\alpha_{0}+\beta X_{\mathrm{ij}}+\sum_{k=1}^{3} \beta_{k}X_{\mathrm{kij}}+\nu_{i}+\eta_{i}+\gamma_{j}$ (Spatial and temporal random effect

terms) *M_2_*

$\log\left( \theta_{ij} \right)=\alpha_{0}+\beta X_{ij}+\sum_{k=1}^{3} \beta_{k}X_{kij}+\nu_{i}+\eta_{i}+\gamma_{j}+\psi_{ij}$ (With Spatial component term, temporal random effect and spatial-temporal interaction effect) *M_3_*

Random effects specifications for the spatial random terms include$\nu_{i}$ and$\eta_{i}$. Here, $\upsilon_{i}$ is the unstructured noise term that follows a normal distribution $\upsilon_{i}\sim N\left( 0,\sigma_{\upsilon}^{2} \right)$ and

$$\eta_{i}|{\eta_{j}}_{,j\neq i}\sim N\left[ \frac{\sum_{j\in\delta_{i}} W_{ij}\eta_{i}}{\sum_{j\in\delta_{i}} W_{ij}},\frac{\sigma_{\eta}^{2}}{\sum_{j\in\delta_{i}} W_{ij}} \right]$$

Where $\delta_{i}$ is the set of villages that adjacent to village *i,* and $W_{ij}$ is the weight of neighboring village *j* and *i*. Weight $W_{ij}$ is considered to be 1 when villages *i* and *j* share the same boundary and 0 otherwise. The prior distribution of the random effect term $\eta_{i}$ is a conditional autoregressive prior used to model the spatial dependence. Model two and three included spatial random effects which arising from a Gaussian stationary process with covariance matrix capturing correlation between any pair of villages as a function of their distance.

The temporal random effect determines the prior distribution represented by $\gamma_{j}$ and we consider a first order auto-regression AR (1) given by $\gamma_{j}=\rho\gamma_{j-1}+\epsilon_{j}$ with partial autocorrelation function, $\epsilon_{j}\sim N(0, \tau^{-1})$ and $\gamma_{j}\sim N(\rho\gamma_{j-1}, \tau^{-1})$. The distribution of the interaction term $\psi_{\mathrm{ij}}$ is characterized by a precision matrix obtained as the Kronecker product of the precisions of $\upsilon_{i}$and $\gamma_{j}$.
