# Supplementary material for: Assessing the effects of mosquito nets on malaria mortality using a space time model: a case study of Rufiji and Ifakara Health and Demographic Surveillance System sites in rural Tanzania
Source: Malar J. 2016 May 4;15:257. doi: 10.1186/s12936-016-1311-9 (PMC4857246; doi:10.1186/s12936-016-1311-9)

**Figure 3: Observed and predicted malaria mortality rate using space time model 2 for 2012 in Ifakara HDSS**


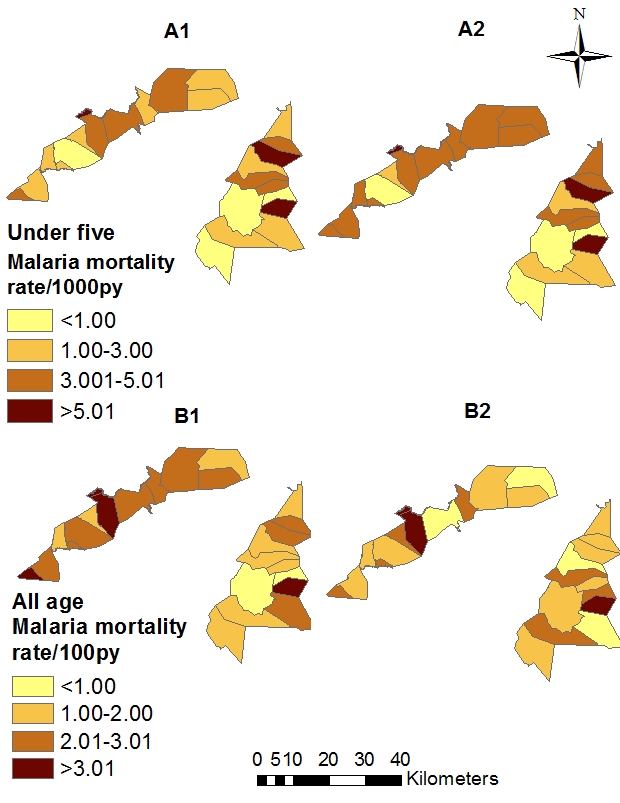

Supplement: Supplementary file 3 — 10.1186/s12936-016-1311-9 Observed and predicted malaria mortality rate using model 2 for 2012in Ifakara HDSS. A1 Observed malaria mortality rate for under-five, A2 predicted malaria mortality rate for under-five, B1 Observed malaria mortality rate for all age and B2 Predicted malaria mortality for all age. [file 12936_2016_1311_MOESM3_ESM.doc]
